# Self-Rated Health and Life Satisfaction among Elderly Migrants in China: A Moderated Mediation Model of Resilience and Upward Intergenerational Support

**DOI:** 10.3390/ijerph192417009

**Published:** 2022-12-18

**Authors:** Li Zhang, Yanjie Hou, Hao Wang, Jun Yao

**Affiliations:** 1 School of Health Economics and Management, Nanjing University of Chinese Medicine, Nanjing 210023, China; 2 School of Health Policy and Management, Nanjing Medical University, Nanjing 211166, China; 3 School of Nursing, Nanjing Medical University, Nanjing 211166, China

**Keywords:** self-rated health, life satisfaction, resilience, upward intergenerational support, elderly migrants

## Abstract

Objective: This study aimed to test whether resilience mediates the association between self-rated health and life satisfaction and the moderated effect of upward intergenerational support among elderly migrants in China. Method: We used self-reported data collected from 654 elderly migrants in Nanjing. The regression analysis using bootstrapping methods was conducted to explore the mediating and moderating effects. Results: Resilience mediated the relationship between self-rated health and life satisfaction among elderly migrants in China. The moderated mediation analysis indicated that the upward intergenerational support moderated the path between self-rated health and resilience. Specifically, compared with those with a low level of emotional support, the self-rated health of elderly migrants with a high level of emotional support has a stronger effect on resilience. Moreover, the self-rated health of elderly migrants with a low level of financial support has a stronger effect on resilience than those with a higher level of financial support. Conclusion: Resilience could partially mediate the effect of self-rated health on life satisfaction among elderly migrants in China, and upward intergenerational support moderated the indirect relationship between self-rated health and life satisfaction via resilience.

## 1. Introduction

Life satisfaction is defined as an individual’s subjective judgment about his/her quality of personal life [1]. In previous studies, life satisfaction has been taken as an indicator of one’s subjective well-being [2]. In the context of urbanization, an increasing number of rural elderlies migrate to cities to live with their children, mainly for grandparenting. Confronted with various challenges in a new environment, this elderly population may develop mental problems that decrease their life satisfaction. Mounting gerontological studies themed on life satisfaction have been conducted in China, but few have analyzed the life satisfaction of elderly migrants. Hence, the factors influencing life satisfaction and their mechanisms should be clarified to design effective interventions to increase the life satisfaction of elderly migrants in China.

### 1.1. Self-Rated Health and Life Satisfaction

Self-rated health is the health status evaluated by an individual with a combination of subjective and objective (not only objective) indicators [3]. For the elderly, improving their physical capacity can increase their life satisfaction [4]. The impact of self-rated health on life satisfaction has been confirmed in recent studies [5,6]. Several studies have found that there is a significantly positive association between self-rated health and life satisfaction [7,8,9]. This association has also been confirmed among Chinese elderly migrants [10]. However, little is known about the potential mechanisms linking them. Thus, in this study, we aimed to screen the factors mediating this association and related mechanisms.

### 1.2. Self-Rated Health, Resilience and Life Satisfaction

Resilience is an individual’s capacity to deal with and recover from significant adversities, such as stress and trauma [11]. The resilience framework thinks that resilience is a dynamic course that can drive a person to grow in the face of adversities [12]. It helps to avoid stress-induced mental disorders, such as depression, posttraumatic stress disorder, and anxiety [13,14]. In past studies, researchers have found that resilience is an important mediating variable, which plays a crucial role in explaining the relationship between some adverse events and physical and mental health. Empirical evidence has demonstrated the association between self-rated health and resilience [15,16,17]. People with better self-rated health tend to have more energy and the ability to deal with life adversities and recover from them. Several cross-sectional studies have revealed a positive association between self-rated health and resilience [18,19,20]. In addition, a longitudinal study has confirmed better self-rated health as the protective factor of resilience after a three-year follow-up [21].

Previous studies have also found a significant association between resilience and life satisfaction. Individuals with a stronger resilience achieve better life satisfaction, appearing more peaceful and happier than those with a weaker resilience [11,22]. Empirical studies reach the consensus that resilience exerts a positive effect on life satisfaction [23,24], and the association is consistent across samples with diverse backgrounds [25]. Based on these findings, some researchers have further investigated the relations among self-rated health, resilience, and life satisfaction [19]. In these studies, resilience is often regarded as a covariable, moderator [26], or a mediator [24,25], and they have found that resilience plays a crucial role in the relationship between self-rated health and life satisfaction. However, no research has examined the mediating role of resilience between self-rated health and life satisfaction among elderly migrants, which may be important in improving elderly migrants’ life satisfaction.

### 1.3. Self-Rated Health, Upward Intergenerational Support, and Resilience

In China, intergenerational support is closely related to the health of the elderly, especially “upward” intergenerational support [27]. Upward intergenerational support refers to the instrumental, financial, or emotional support that elderly parents receive from their adult children. A cross-sectional survey has shown that intergenerational support from children has a significant effect on the self-rated physical health and psychological health of elderly parents in rural China [28]. Lower emotional or instrumental support from children reduces the level of self-rated health and increases depressive symptoms in older adults [29]. Among all combinations of support, emotional support is the most effective in improving parents’ psychological health [30]. Older adults who receive higher levels of support from children in processing household chores tend to experience improvement in self-rated health [31]. Older adults who receive intergenerational support have higher levels of self-rated health, which consequently enhances mental and physical resilience. In other words, the direct relationship between self-rated health and resilience is stronger in elderly migrants with high levels of upward intergenerational support. However, no previous studies have examined whether upward intergenerational support is a promoting factor that moderates the relationship between self-rated health and resilience.

### 1.4. The Present Study

**Figure 1:**
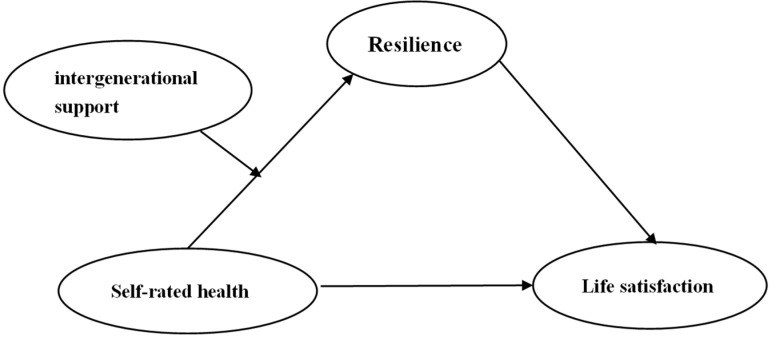
Moderated mediation model predicting life satisfaction.

In the current study, we first tested whether resilience mediates the relationship between self-rated health and life satisfaction among elderly migrants in China. Second, we explored whether upward intergenerational support moderates the association between self-rated health and resilience. Based on the literature review, we proposed the following hypotheses developed the model (Figure 1):

**Hypothesis 1.**  *Self-rated health is positively correlated with life satisfaction*.

**Hypothesis 2.**  *Resilience mediates the relationship between self-rated health and life satisfaction.*

**Hypothesis 3.**  *The mediation path from self-rated health to life satisfaction via resilience is moderated by upward intergenerational support*.

## 2. Methods

### 2.1. Participants and Sampling

This research was carried out in Nanjing (Jiangsu, China) in 2020. Nanjing has a developed economy, with the Gross Domestic Product per capita of about 159,300 (RMB, yuan) in 2020. Included were the elderly migrants aged 60 years and above who had moved to live in Nanjing for no more than 10 years and had not changed their Hukou (household registration in China) to Nanjing.

This survey randomly selected 7 districts in Nanjing (Qinhuai, Qixia, Gulou, Xuanwu, Jianye, Yuhuatai, and Jiangning District), then randomly selected 3 communities in each district, and finally recruited elderly migrants who met the inclusion criteria in these 21 communities. All participants were informed of the purpose of the study and volunteered to participate in, and a total of 654 valid questionnaires were obtained.

### 2.2. Measures

An independent variable was self-rated health, and a dependent variable was life satisfaction. The study also assessed resilience as a mediating variable and intergenerational support as a moderating variable. Control variables included age, gender, education level, and personality.

#### 2.2.1. Self-Rated Health

Self-rated health was assessed by an 8-item short-form survey (SF-8) in two dimensions. Four items, including general health (GH), physical function (PF), role physical (RP), and bodily pain (BP), were measured to indicate physical component summary (PCS). The other 4 items, including Energy/fatigue (vitality, VT), social functioning (SF), role limitations due to emotional problems (role emotional, RE), and psychological distress and well-being (mental health, MH), were measured to indicate mental component summary (MCS) [32,33]. Each of those eight items was written into a question that was asked to be answered within four weeks. We recorded the responses on five or six-point Likert scale for the eight items. For both dimensions, a higher score indicated better health. Alternate form reliability for the eight items ranged from 0.70 to 0.88. In this study, Cronbach’s alpha was 0.862, indicating good reliability of SF-8 survey.

#### 2.2.2. Life Satisfaction

Life satisfaction was assessed by the Satisfaction with Life Scale (SWLS) including 5 items [34] (“in most ways my life is close to my ideal”, “the conditions of my life are excellent”, “I am satisfied with my life”, “so far I have gotten the important things I want in my life”, “if I could live my life over, I would change almost nothing”), with 7 choices available for each item rated on a scale of 1 (strongly degree) to 7 (strongly disagree). The total score ranged from 5 to 35. A higher score indicated a higher degree of life satisfaction. In this sample, Cronbach’s alpha was 0.911, demonstrating good internal reliability of the scale.

#### 2.2.3. Resilience

Resilience was measured by a 10-item Connor–Davidson Resilience Scale (CD-RISC-10). The scale was composed of 10 items: “can adapt to change”, “can deal with whatever comes”, “can see humorous side of problems”, “can cope with stress can strengthen me”, “can recover from illness or hardship”, “can achieve goals despite obstacles”, “can stay focused under pressure”, “cannot be easily discouraged by failure”, “can think of self as strong person”, “can handle unpleasant feelings” [35]. Respondents rated these items on a 5-point scale from 1 (not true at all) to 5 (true nearly all the time) [36]. The total score ranged from 10 to 50. A higher score indicated stronger resilience. Cronbach’s alpha was 0.922, indicating good reliability of the sample.

#### 2.2.4. Intergenerational Support

Intergenerational support was assessed in three dimensions: emotional, financial, and instrumental support. Emotional support was upward, referring to the support that the elderly migrants received from their children. Financial support was upward, and instrumental support was downward, both referring to intergenerational support.

Emotional support was measured by three questions based on the Intergenerational Solidarity Inventory [37]: (a) “Did you feel emotionally close to your child?”; (b) “Is this child willing to communicate with you in terms of your worries and troubles?”; and (c) “Did you get along with this child?” [38]. For each question, the answers were scored from 0 to 2 (0 = not at all, 1 = somewhat, 2 = very much). The summed score represented the level of emotional support (range = 2–8; Cronbach’s alpha = 0.73).

Financial support was assessed by two questions: “How much financial support did you provide your child in the past 12 months?”, “How much financial support did you receive from your child in the past 12 months?” [39]. The answer was rearranged into two categories: 0 = <10,000 RMB, 1 = ≥10,000 RMB.

Instrumental support was assessed by two questions: “How often have you helped the child do housework or care tasks in the past 12 months, such as cleaning, washing and taking care of grandchildren?”, “How often has this child helped you do housework or care tasks in the past 12 months?” [40,41]. The answer was rearranged into two categories: 0 = none or occasionally, 1 = often.

#### 2.2.5. Sociodemographic Factors

Age (60–64, 65–69, 70–74, 75+ years), gender (0 = female, 1 = male), education level (0 = illiteracy, 1 = elementary school, 2 = junior middle school, 3 = senior middle school, 4 = college and above), yearly income (0 = less than 5000 RMB, 1 = 5000–9999, 2 = 10,000–19,999, 3 = 20,000–39,999, 4 = 40,000–79,999, 5 = 80,000–119,999, 6 = more than 120,000 RMB) and Hukou (0 = urban, 1 = rural) were included as control variables in all models in this study because they had potential influence to other variables.

### 2.3. Data Analysis

In this study, descriptive statistics and distributions were examined for all the variables. Harman’s one-way test and confirmatory factor analysis were used to evaluate method bias. Pearson’s correlation coefficient was used to verify the correlation between variables. The hierarchical linear regression analyses were performed to test the mediating effect of resilience. All analyses were run on SPSS 23 PROCESS plugin was used to analyze the mediating and moderating effects. PROCESS is an observed variable OLS and logistic regression path analysis modeling tool. It is widely used through the social, business, and health sciences for estimating direct and indirect effects in single and multiple mediator models (parallel and serial), two- and three-way interactions in moderation models, along with simple slopes and regions of significance for probing interactions, and conditional indirect effects in moderated mediation models with a single or multiple mediators or moderators (The PROCESS macro for SPSS, SAS and R).

## 3. Results

### 3.1. Sample Characteristics

Table 1 shows the sociodemographic characteristics of the sample. Of the 654 participants, most were women (67%), aged 60–70 years (78.1%), had a lower income (38.1%), a rural Hukou (69.3%), a married status (84.6%), and an education level of elementary school and below (54.1%). Most of them were working (88.2%) and had migrated to Nanjing to care for their children and grandchildren (77.4%).

### 3.2. Bivariate Relationships among Key Variables

Table 2 presents the descriptive variables. Self-rated health correlated strongly with resilience and life satisfaction, and resilience correlated strongly with life satisfaction.

Emotional support and financial support correlated strongly with resilience, and instrumental support (downward) also correlated with resilience, but instrumental support (upward) did not correlate with resilience.

### 3.3. Mediation Analyses

In testing our first and second hypotheses, a mediational analysis was conducted by PROCESS Model 4. As shown in Table 3, self-rated health was directly associated with resilience (β = 0.377, *p* < 0.01), and resilience was directly associated with life satisfaction (β = 0.307, *p* < 0.01). The association between self-rated health and life satisfaction was also significant (β = 0.217, *p* < 0.01), which can confirm our first hypothesis. We also found a significant indirect effect of self-rated health on life satisfaction via resilience. The bootstrapping results indicated an indirect effect (β = 0.116, 95%CI: [0.081, 0.154]). The indirect effect accounted for 34.9% of the total effect, suggesting that resilience played an evident mediating role in the association between self-rated health and life satisfaction. These findings supported our second study hypothesis.

### 3.4. Moderated Mediation Analyses

In order to test our third hypothesis, a moderated mediation analysis was conducted by PROCESS Model 7. As intergenerational support has three types, we set five moderator variables in the models, including emotional support, financial support (upward and downward), and instrumental support (upward and downward). Table 4 shows the results of the moderated mediation analysis. The effect of the interaction between self-rated health and emotional support on resilience was positive and statistically significant (β = 0.086, *p* < 0.05). In terms of bi-directional financial and instrumental support, only the interaction between self-rated health and financial support (upward) had a significant negative effect (β = −0.222, *p* < 0.05) on resilience among elderly migrants [41].

**Figure 2:**
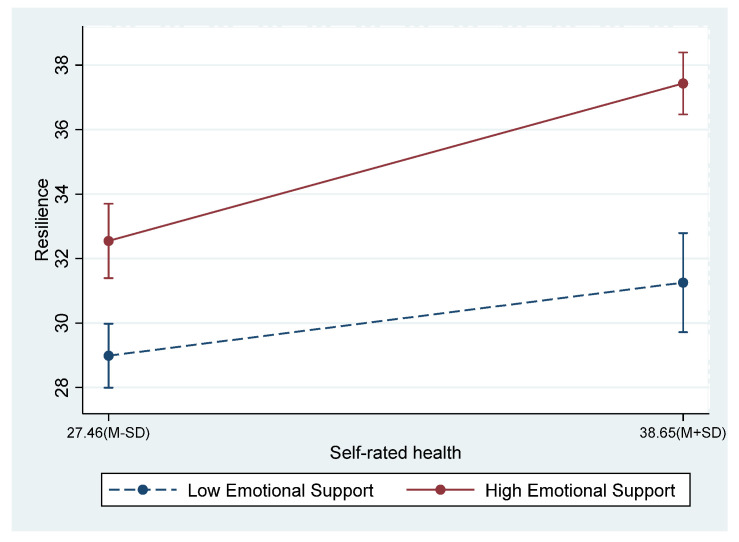
Emotional support moderated the effect of self-rated health on resilience.

**Figure 3:**
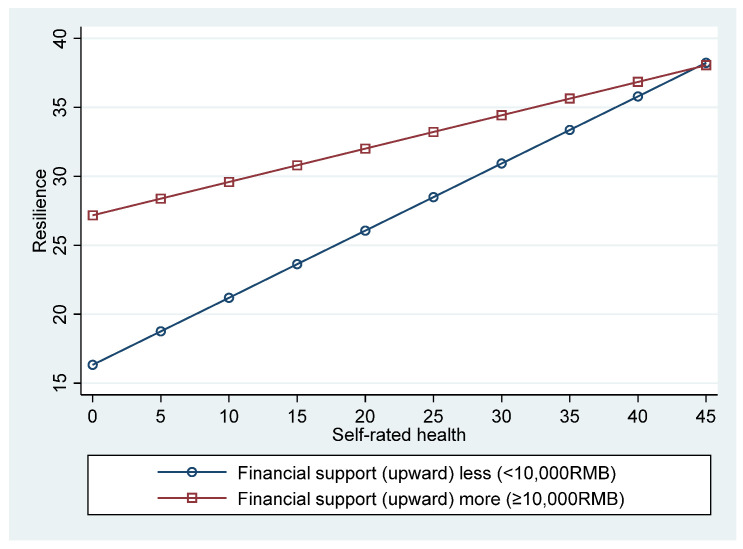
Financial support (upward) moderated the effect of self-rated health on resilience.

Figure 2 illustrates the interaction at high (M + SD) and low (M − SD) levels of self-rated health and emotional support. The plots show the interaction between self-rated health and emotional support on resilience, suggesting a stronger positive association between self-rated health and resilience in the elderly migrants at a higher emotional support level compared with those at a lower emotional support level. Figure 3 illustrates the effect of the interaction between self-rated health and financial support (upward) on resilience. The plots show the interaction between self-rated health and financial support (upward), suggesting a negative association between self-rated health and resilience in the elderly migrant at a higher level of upward financial support (≥10,000 RMB), compared with those at a lower level (<10,000 RMB).

In addition, the moderated mediation indexes [42] were also calculated. Table 4 shows the total moderated mediation effect. When emotional support was used as a moderator, the indirect effect was significant (95%CI: 0.003, 0.046), indicating that the indirect effect of self-rated health and life satisfaction through resilience was moderated by emotional support, and when financial support (upward) was used as a moderator, the indirect effect was also significant (95%CI: −0.123, −0.015), indicating that the indirect effect of self-rated health and life satisfaction through resilience was moderated by financial support (upward).

## 4. Discussion

In the present study, we verified that resilience plays a mediating role in the relationship between self-rated health and life satisfaction among elderly migrants in China. As expected, high levels of self-rated health and resilience were positively associated with life satisfaction. Furthermore, we also revealed the partial mediating effects of resilience on the path from self-rated health to life satisfaction. Moreover, upward intergenerational support moderated the indirect relationship between self-rated health and life satisfaction via resilience.

### 4.1. Self-Rated Health and Life Satisfaction

In line with Hypothesis 1, better self-rated health was associated with a higher level of life satisfaction among the elderly migrants. A plausible explanation for this finding is that elderly migrants with better self-rated health have stronger health self-efficacy. Health self-efficacy can improve the self-confidence of elderly migrants to cope with setbacks so as to improve their life satisfaction. The main reasons for their migration in this study include grandparenting and decreasing the life pressure of their children [43]. Moreover, most of the elderly migrate from rural areas, with a low education level and less knowledge about basic public health services. Most of them have not even established their health records [44] and have poor health consciousness [45]. On the other hand, due to the difference in healthcare policies between regions [46], many elderly migrants do not seek treatment actively when they become sick. As a result, the utilization rate of medical services is low among elderly migrants. If they become sick and have to be cared for by their children, they may lose self-efficacy and even develop psychological disorders, such as depression and anxiety, that can reduce life satisfaction. In China, the community has the responsibility to establish health records or cards for elderly migrants so as to monitor their health status [47]. Medical policies have been released to reduce inequality in public health services between regions [43].

### 4.2. Mediating Role of Resilience

This study found that resilience could partially mediate the effect of self-rated health on life satisfaction among elderly migrants, which verifies our Hypothesis 2 and echoes the conclusions of other researchers [19]. This indicates that even if the elderly migrants have poor self-rated health, their resilience still helps them to cope with the negative factors of life satisfaction. Previous studies have shown that elderly migrants are at a higher risk of health problems than local-resident elderlies [48,49,50]. For instance, they are vulnerable to residential segregation, communicable and sexually transmitted diseases, policy inequality, and occupational injuries and diseases [49,51]. Their health status decreases with the increase in age and flow dilemma. However, psychological resilience can protect them from the sense of maladaptation caused by poor physique, pressure, and frustration and maintain a good mental state. A strong resilience is conducive for the elderly migrants to take active actions to adjust their physical and mental state. Physical and mental health also has a great effect on life satisfaction. The present study showed that life satisfaction is more affected by the resilience to cope with adversities in elderly migrants. Therefore, effective efforts should be taken to enhance the resilience of elderly migrants in China.

### 4.3. Moderating Role of Upward Intergenerational Support

We found that the mediation path from self-rated health to life satisfaction via resilience was moderated by upward intergenerational support, which proves Hypothesis 3. A higher level of upward intergenerational support predicted a greater effect of self-rated health on resilience. Our results supported the resource conservation theory of Hobfoll [52]. Moreover, compared with that in the elderly migrants at a lower level of emotional support, the self-rated health had a significantly stronger effect on resilience in those at a higher level, and compared with that in the elderly migrants at a higher level of financial support, the self-rated health had a significantly stronger effect on resilience in those at a lower level.

For the elderly having migrated into a new environment, failure of social integration negatively affects their self-rated health and life satisfaction [53,54]. One’s active adaptation to old age directly depends on his/her ability to acquire resources [55], which in turn affects the level of physical health and resilience. Emotional support from their children can reduce loneliness and depression in elderly migrants [56] and enhance their abilities to cope with loneliness, depression, anxiety, and other negative emotions [30,57]. Financial support from children is the main source of income for elderly migrants. However, excessive financial support may pose a psychological burden on the elderly. Therefore, appropriate financial support is needed to strengthen the ability of elderly migrants to acquire medical services [58] and improve their physical health. In general, the negative effects of age and migration on the self-rated health of elderly migrants can be buffered by upward generational support, either financial or emotional. A harmonious intergenerational relationship is needed to guarantee this support.

## 5. Conclusions

Our study shows that self-rated health is positively correlated with life satisfaction among elderly migrants in China; resilience mediates the relationship between self-rated health and life satisfaction; the mediation path from self-rated health to life satisfaction via resilience is moderated by upward intergenerational support. Given these conclusions, we make the following recommendations. First of all, the government should pay attention to the health level of elderly migrants, set up mechanisms to let them enroll in the family doctor contract service in urban communities, improve access to medical services in different places and make it easier for these people to seek medical treatment in cities, improve the policies about the off-site medical treatment which can make the elderly migrants receive medical treatment conveniently. Secondly, the children should strengthen their emotional communication with their parents, care about them, and often chat with them instead of giving them more money, making the elderly migrants feel more emotional support, which is good for improving elderly migrants’ life satisfaction.

## 6. Limitation and Future Studies

First, this is a cross-sectional survey, which cannot find the causal relationships between variables. In the future, we will conduct longitudinal studies focusing on elderly migrants’ life satisfaction. Second, some other variables may mediate the association between self-rated health and life satisfaction, such as loneliness, anxiety, depression, etc. In future studies, we will further analyze the effects of these variables. Lastly, all the participants were recruited from Nanjing, Jiangsu, China. Multi-center studies are needed in the future to examine the findings of this study.

## Figures and Tables

**Table 1:** Participant Sociodemographic Characteristics.

Variables		Frequency (Number of Participants)	Percent of Sample (%)
Gender	Male	216	33.0
	Female	438	67.0
Age	60–64	282	43.1
(years)	65–69	229	35.0
	70–74	102	15.6
	≥75	41	6.3
Yearly income	<5000	249	38.1
(RMB, yuan)	5000–9999	126	19.3
	10,000–19,999	87	13.3
	20,000–39,999	112	17.1
	40,000–79,999	59	9.0
	>80,000	21	3.2
Hukou	Urban	453	69.3
	Rural	201	30.7
Marital status	Married	553	84.6
	Widow	90	13.8
	Divorced	11	1.7
Education level	Illiteracy	167	25.5
	Elementary school	187	28.6
	Junior middle school	158	24.2
	High middle school	104	15.9
	College and above	38	5.8
Working status	Working	77	11.8
	Not working	577	88.2
Migration reason	Caring for children or grandchildren	506	77.4
	Living the life in retirement	86	13.1
	Working	19	2.9
	Others	43	6.6

**Table 2:** Bivariate correlations among key variables.

	M	SD	1	2	3	4	5	6	7	8
1. Self health	33.054	5.592	1							
2. SWLS	26.740	5.372	0.367 ***	1						
3. RISC	33.064	7.527	0.330 ***	0.514 ***	1					
4. Emotion	9.353	1.406	0.307 ***	0.404 ***	0.335 ***	1				
5. Financial_d	0.116	0.321	0.113 ***	0.018	0.107 ***	0.045	1			
6. Financial_u	0.255	0.436	0.095 **	0.162 ***	0.181 ***	0.180 ***	0.083 **	1		
7. Instrumental_d	0.726	0.446	0.138 ***	0.254 ***	0.350 ***	0.196 ***	0.051	0.139 ***	1	
8. Instrumental_u	0.480	0.500	0.015	0.031	−0.016	0.109	−0.005	−0.022	0.055	1

*** *p* < 0.01; ** *p* < 0.05; M, mean scores; SD, standard deviation; Self health, self-rated health; SWLS, life satisfaction; RISC, Resilience; Emotion, emotional support (upward); Financial_d, financial support (downward); Financial_u, financial support (upward); Instrumental_d, instrumental support (downward); Instrumental_u, instrumental support (upward).

**Table 3:** Mediation analysis results about the effect of self-rated health on life satisfaction.

	β	SE	t	LLCI	ULCI	R2
Outcome variable: RISC						
constant	6.653	4.510	1.475	−2.204	15.510	0.194
Self-rated health (X)	0.377	0.049	7.632 ***	0.280	0.475	
Outcome variable: SWLS						
constant	3.652	2.973	1.229	−2.185	9.490	0.316
Self-rated health (X)	0.217	0.034	6.377 ***	0.150	0.283	
RISC (M)	0.307	0.026	11.865 ***	0.256	0.358	
Effect of X→M→Y						
Total effect	0.332	0.036	9.266 ***	0.262	0.403	
Direct effect	0.217	0.034	6.377 ***	0.150	0.283	
Indirect effect (RISC)	0.116	0.019		0.081	0.154	

β, standardized coefficients; SE, standard error; LLCI and ULCI, lower level and upper level of the bias-corrected 95% bootstrap confidence interval; X, self-rated health; Y, life satisfaction; M, resilience; *** *p* < 0.01.

**Table 4:** Moderated mediation analysis results about the effect of self-rated health on life satisfaction.

	β	SE	t	LLCI	ULCI	R^2^
Outcome variables: RISC						
constant	19.382	3.924	4.939 ***	11.677	27.088	0.243
Self-rated health	0.295	0.050	5.909 ***	0.197	0.393	
W1	1.300	0.205	6.332 ***	0.897	1.704	
Self-rated health × W1	0.086	0.033	2.554 **	0.020	0.151	
constant	18.563	4.071	4.560 ***	10.569	26.558	0.199
Self-rated health	0.382	0.049	7.737 ***	0.285	0.479	
W2_d	1.675	0.835	2.006 **	0.035	3.315	
Self-rated health × W2_d	0.082	0.153	0.357	−0.218	0.382	
constant	19.032	3.967	4.797 ***	11.242	26.823	0.227
Self-rated health	0.352	0.049	7.218 ***	0.256	0.448	
W2_u	3.025	0.607	4.982 ***	1.833	4.218	
Self-rated health × W2_u	−0.222	0.108	−2.050 **	−0.434	−0.009	
constant	1.848	4.888	0.304	−8.113	11.082	0.273
Self-rated health	0.460	0.092	4.981 ***	0.279	0.642	
W3_d	10.358	3.472	2.983 ***	3.541	17.176	
Self-rated health × W3_d	−0.173	0.106	−1.630	−0.381	0.035	
constant	6.157	4.870	1.264	−3.406	15.719	0.194
Self-rated health	0.396	0.068	5.841 ***	0.263	0.530	
W3_u	0.976	3.208	0.304	−5.324	7.275	
Self-rated health × W3_u	−0.038	0.096	−0.402	−0.226	0.149	
Outcome variables: SWLS						
constant	3.652	2.973	1.229	−2.195	9.490	0.316
Self-rated health	0.217	0.034	6.377 ***	0.150	0.283	
RISC	0.307	0.026	11.865 ***	0.256	0.358	
Direct effect of X on Y						
	0.217	0.034	6.377	0.150	0.283	
Index of moderated mediation						
Emotional support	0.026	0.011		0.003	0.046	
Financial support (upward)	−0.068	0.028		−0.123	−0.015	

*** *p* < 0.01; ** *p* < 0.05; W1: emotional support; W2_d: Financial support (downward); W2_u: Financial support (upward); W3_d: Instrumental support (downward); W3_u: Instrumental support (upward).

## Data Availability

The datasets used and analysed in this study are available from the corresponding author on reasonable request.
